# Short stature and *SHOX* (Short stature homeobox) variants—efficacy of screening using various strategies

**DOI:** 10.7717/peerj.10236

**Published:** 2020-11-17

**Authors:** Pavlina Capkova, Zuzana Capkova, Peter Rohon, Katerina Adamová, Jirina Zapletalova

**Affiliations:** 1 Department of Medical Genetics, University Hospital Olomouc, Olomouc, Czech Republic; 2 Department of Medical Genetics, Faculty of Medicine and Dentistry, Palacky University Olomouc, Olomouc, Czech Republic; 3 Department of Pediatrics, Faculty of Medicine and Dentistry, Palacky University Olomouc, Olomouc, Czech Republic

**Keywords:** SHOX, Short stature, Leri-Weill dyschondrosteosis, Turner syndrome, Screening for mutations, Idiopathic short stature, MLPA, FISH, Karyotyping, Sequencing

## Abstract

**Background:**

*SHOX* mutations have previously been described as causes of Léri-Weill dyschondrosteosis (LWD), Langer mesomelic dysplasia (LMD), and idiopathic short stature. The loss of X chromosome—Turner syndrome or mosaic 45,X/46,XX or 46,XY—also leads to the heterozygous loss of *SHOX* in patients with short stature only or with features similar to LWD. The aim of this study was to assess the efficacy of the targeted screening for *SHOX* variants, which involved different methods in the laboratory analysis of short stature. We determined the significance and positive predictive value of short stature for the detection of *SHOX* variants.

**Methods:**

Targeted screening for variants in *SHOX* involving MLPA, sequencing, karyotyping and FISH was performed in the short stature cohort (*N* = 174) and control cohort (*N* = 91). The significance of short stature and particular characteristics for the detection of *SHOX* variants was determined by Fisher’s exact test, and the probability of *SHOX* mutation occurrence was calculated using a forward/stepwise logistic regression model.

**Results:**

In total, 27 and 15 variants influencing *SHOX* were detected in the short stature and control cohorts, respectively (*p* > 0.01). Sex chromosome aberrations and pathogenic CNV resulting in diagnosis were detected in eight (4.6%) and five (2.9%) patients of the short stature group and three (3.3%) and one (1.1%) individuals of the control group. VUS variants were discovered in 14 (8.0%) and 11 (12.1%) individuals of the short stature and control groups, respectively. MLPA demonstrated the detection rate of 13.22%, and it can be used as a frontline method for detection of aberrations involving *SHOX*. However, only mosaicism of monosomy X with a higher frequency of monosomic cells could be reliably discovered by this method. Karyotyping and FISH can compensate for this limitation; their detection rates in short stature group were 3.55% and 13.46% (*N* = 52), respectively. FISH proved to be more effective than karyotyping in the study as it could reveal cryptic mosaics in some cases where karyotyping initially failed to detect such a clone. We suggest adding FISH on different tissue than peripheral blood to verify sex-chromosome constitution, especially in cases with karyotypes: 45,X; mosaic 45,X/46,XX or 46,XY; 46,Xidic(Y) detected from blood; in children, where mosaic 45,X was detected prenatally but was not confirmed from peripheral blood. The correlation of short stature with the occurrence of *SHOX* mutations was insignificant and short stature demonstrates a low positive predictive value-15.5% as unique indicator for *SHOX* mutations. The typical skeletal signs of LWD, including Madelung deformity and disproportionate growth, positively correlate with the findings of pathogenic *SHOX* variants (*p* < 0.01) by Fisher’s exact test but not with the findings of VUS variants in *SHOX* which are more prevalent in the individuals with idiopathic short stature or in the individuals with normal height.

## Introduction

### Background

Growth retardation, a common condition leading to reduced height, is defined as the deviation of an individual’s height of more than two standard deviation score (SDS) below the mean in the population or the estimated familial target height (Amin, Mushtaq & Alvi, 2015). Short stature can be caused by nongenetic factors, such as nutrition, chronic systemic disorders, and emotional or psychosocial deprivation. Most forms of short stature, however, are based on genetic causes (Turner syndrome, Leri Weill dyschondrosteosis, Langer mesomelic dysplasia) (Seaver, Irons & American College of Medical Genetics Professional Practice and Guidelines Committee, 2009). *SHOX* gene mutations have previously been described as causes of Leri-Weill dyschondrosteosis (LWD), Langer mesomelic dysplasia (LMD), idiopathic short stature (ISS), and its haploinsufficiency is described as the cause of growth restriction in Turner syndrome (TS) (Belin et al., 1998; Rao et al., 1997; Benito-Sanz et al., 2005; Benito-Sanz et al., 2006; Benito-Sanz et al., 2011; Hirschfeldova et al., 2012; Zinn et al., 2002; Campos-Barros et al., 2007; Rappold et al., 2002). A wide spectrum of *SHOX* variants have been identified so far. However, not all can be directly associated with short stature in patients. ISS is a condition in which the height of the individual is more than 2 standard deviation (SD) below the corresponding mean height for a given age, sex, and population, and in whom no identifiable disorder is present (Wit et al., 2008). Heterozygous mutations of *SHOX* and/or its regulatory elements are detected in approximately 70% of LWD patients and involve 70–80% large deletions, 2–6% partial deletions, 20–25% point mutations (Benito-Sanz et al., 2005; Benito-Sanz et al., 2006; Benito-Sanz et al., 2011; Binder, 2011; Caliebe et al., 2012). Homozygous or compound heterozygous mutations of *SHOX* and/or its downstream enhancers are detected in 75% of LMD patients (Benito-Sanz et al., 2006; Benito-Sanz et al., 2012; Chen et al., 2009; Huber et al., 2006). In patients with ISS, the prevalence of SHOX mutations varies from 2–15% depending on other clinical features and the technologies used (Benito-Sanz et al., 2006; Benito-Sanz et al., 2012; Chen et al., 2009; Huber et al., 2006). However, the number of detected variants including intronic mutations influence the splicing of *SHOX* either with unambiguous or unclear significance for linear growth of individuals in the group of children with short stature is still growing (Thomas et al., 2009; Alharthi et al., 2017; Sandoval et al., 2014; Durand et al., 2011). Chromosomal abnormalities in the sex chromosomes that lead to the heterozygous deletion of *SHOX* are a cause of short stature in patients with TS or patients with ISS (Oliveira & Alves, 2011). There are also similarities to skeletal markers in TS and LWD (Soucek et al., 2013). Duplications have also been reported in LWD and ISS patients (Benito-Sanz et al., 2011).

### Clinical significance

GH therapy is recommended for patients with LWD/TS (Blum et al., 2013; Lebl & Zapletalova, 2011; Amin, Mushtaq & Alvi, 2015). That is why the diagnosis of the syndromes is of great importance, especially in early childhood. The finding of an optimal balance between cost and effectivity of the testing in the population of children with short stature is still under debate especially in the children with ISS (Cohen et al., 2008; Sisley et al., 2013; Collett-Solberg et al., 2019). This group of the patients is heterogenous and growth restriction is often isolated. Determination of the causes of growth failure in the patients is a challenge for clinicians as other symptoms typical for LWD might manifest later in childhood or during puberty e.g., Madelung deformity (Binder, 2011; Fukami et al., 2004). However, GH treatment should start before puberty initialization. Several clinical prediction rules based on multiple anthropometric measurements (Rappold et al., 2007) or the seated height-to standing height ration (Binder, 2011) have been suggested to select ISS patients who have a higher probability of having *SHOX* variants. However, none of these criteria has a high positive predictive value, and clinical utility of the systems is limited by the highly variable clinical presentation of *SHOX* deficiency (Dauber, Rosenfeld & Hirschhorn, 2014). Thus, exclusion of the mutations in *SHOX* is usually indicated in clinical settings based only on short stature. Most textbooks and the previous GRS consensus on the topic of short stature recommend routine laboratory screening for occult disease in asymptomatic short children (Cohen et al., 2008). Karyotyping is a standard technique for exclusion of sex chromosome aberrations, namely TS (including mosaic), and MLPA and sequencing are tools for detection of subtle changes in the *SHOX* gene. However, even among the patients with mosaic 45,X/46,XX/46,XY we can find very mild phenotypes which actually do not suggest aberration of sex chromosomes. So our hypothesis was that the height might be good biomarker for detection of any variants in *SHOX*. We used a testing algorithm in children with primarily short stature for three years. We performed extensive screening involving the above mentioned methods to assess how effective this screening is if short stature is a “stand alone” predictor for *SHOX* mutations and how effective the particular methods are.

## Materials & Methods

### Aim of study

The study design is retrospective. The aim of the study was to assess the efficacy of screening testing using karyotyping, FISH, MLPA, and Sanger sequencing to detect mutations of the *SHOX* gene in children with short stature (cut off -2SDS). We assessed the positive predictive value of short stature for detection of *SHOX* aberrations. We assessed the significance of particular characteristics (facial dysmorphia; Madelung deformity; skeletal LWD markers—disproportionate growth/mesomelia/rhizomelia/wrist changes/shortening of the fourth and fifth metacarpals/tibial bowing/muscular hypertrophy; heart malformations; other congenital malformations; hypospadia, hypogonadism; micrognathia; neurodevelopmental disorders; microcephaly; macrocephaly; history of IUGR/SGA and family history of short stature) in the detection of *SHOX* mutations/variants.

### Participants

Patients with short stature (deviation more than -2SDS) (*N* = 174) (age: 2–19 years; mean = 8.6; *F* = 101, *M* = 73) as well as a control cohort (deviation less than -2SDS) (*N* = 91) (age: 3–19 years; mean = 8.2; *F* = 55; *M* = 36) of Caucasian ethnicity were recruited from the Department of Medical Genetics and the Department of Pediatrics, University Hospital Olomouc. Endocrine and metabolic disorders were excluded before genetic diagnostic testing was performed. Height measurements and calculations of *Z*-scores, as well as anthropometric measures to determine disproportionate short stature (based on anthropometric measurements—sitting height/height), mesomelia, rhizomelia, etc. were performed at the first visit. Bone age was estimated from X-ray stay together. Disproportionate growth was based on measurement of sitting height. Whenever family members were tested, the results were applied to the short stature group or control cohort according to their height. Signed informed consent was obtained from all participants. Clinical data of the patients were collected from medical records. General observations of the characteristics/comorbidities in patients were made by a geneticist or paediatrician. Apart from growth restrictions, other characteristics/comorbidities were scored: facial dysmorphia; Madelung deformity; skeletal LWD markers—disproportionate growth/mesomelia/rhizomelia/wrist changes/shortening of the fourth and fifth metacarpal bones/tibial bowing/muscular hypertrophy; microcephaly; macrocephaly; heart malformations; other congenital malformations—heart/renal/urogenital/brain; micrognathia; neurodevelopmental disorders; history of IUGR/SGA and family history of short stature. The control cohort consisted of children and teenagers with deviations smaller than -2SDS or patients tested by MLPA, FISH, or karyotyping for different conditions (*SHOX* variant was detected as incidental finding) and adults (parents; volunteers involving laboratory staff and students) with deviations smaller than -2SDS. As we primarily focused on the height as a biomarker for the detection of *SHOX* variants, we used the same criterion for the control/tested group regardless of other symptoms. In volunteers and children tested for different conditions, the DNA samples were anonymized. Signed informed consent was obtained from all participants or their legal representative. All procedures were conducted in accordance with the Declaration of Helsinki. The study was approved with the ethical committee of University Hospital Olomouc (NU20-07-00042 and SUG 87-82).

### Methods

**Figure 1:**
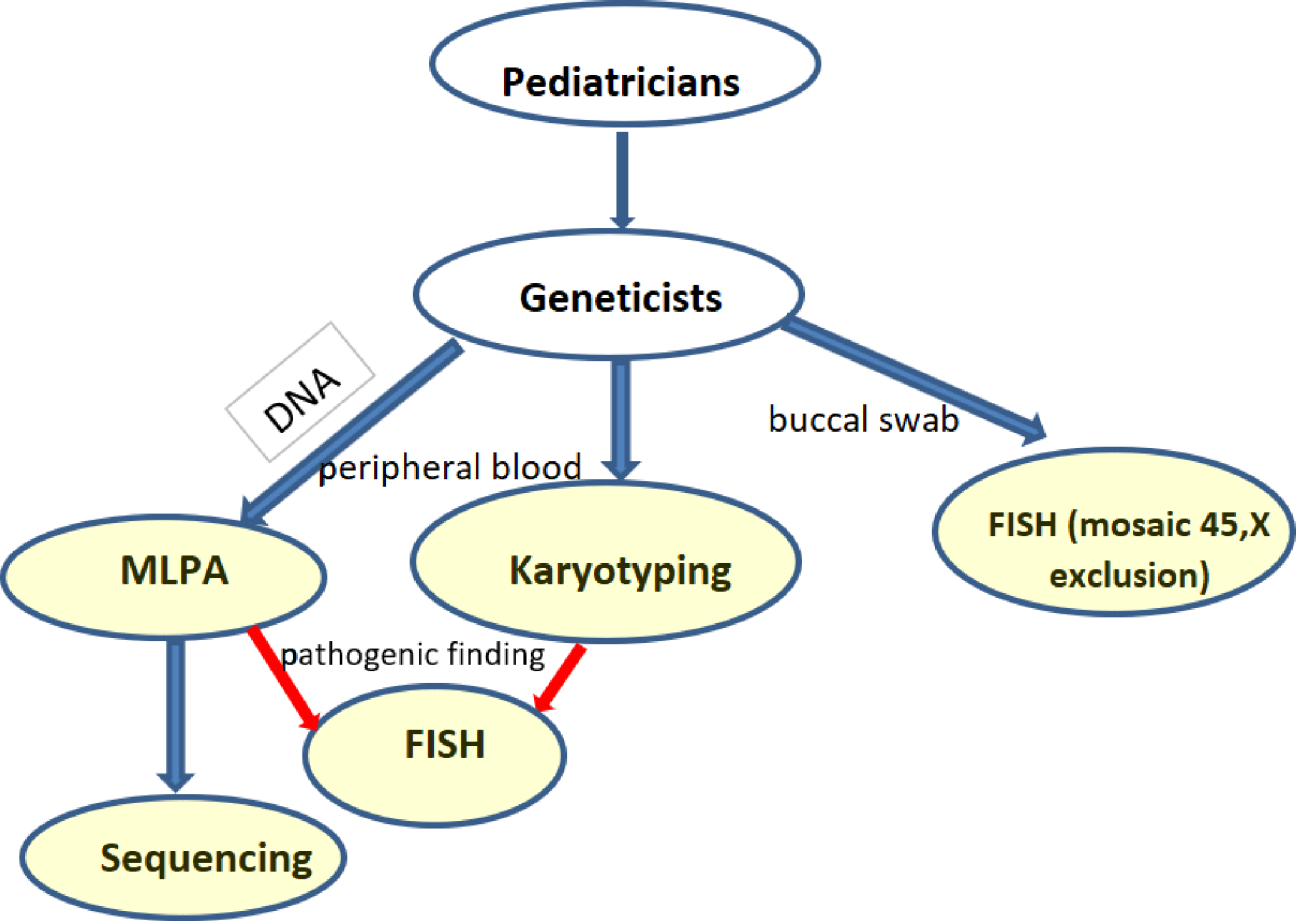
Flowchart of the performed investigations in short stature patients. (blue arrows—routine testing, red arrows—additional testing in case of a pathogenic finding).

Screening for *SHOX* aberrations involved karyotyping (169 short stature group and 49 control), MLPA (174 short stature group and 86 control), Sanger sequencing (154 short stature group and 25 control), and FISH (52 short stature group and 11 control) on buccal smears in relevant cases. The flow chart shows the scheme of the screening (Fig. 1). Cytogenetic analysis was performed using cultured lymphocytes by conventional G-banding with a resolution of 550 bphs. I-FISH on buccal smears was performed with an alpha satellite X,Y –Satellite Enumeration Probes (Cytocell, Cambridge, UK) on interphase nuclei to exclude mosaic of monosomy X in relevant cases. Confirmation of idic(Y) and i(X) were performed with *SHOX* probes (Cytocell, Cambridge, UK). At least 100 cells were checked. A cut-off value was calculated for the healthy control for particular tissues, genders, and probes as mean +3SDS. Where it was suitable, a microarray (Agillent, Affymetrix) was added to determine the extent of gains and losses. The genomic DNA was isolated from peripheral blood using the saline method. MLPA (Multiplex Ligation-Dependent Probe Amplification) was performed with the probemix SALSA MLPA P018-G1 (*SHOX*) MRC Holland. Fragment analysis was performed using an ABI Prism 3130 automated sequencer. Peak areas were assessed by Gene Mapper software (ABI Prism) and the resulting ratio was calculated using Coffalyser software (MRC, Holland). We double checked (two runs) the variants or verified them with another probemix (P070, P036). Sequence analysis of the coding regions was performed by Sanger sequencing: exons 2–6a, 6b, and the nearest intronic flanking codons. The sequence of the primers was as follows: 2R GTGCACAGCGAGGGGC, 2F ACGGGCCGTCCTCTCC, 3R CGTCTCCAAAAGTCCAGGAACC, 3F GAGTATCCTCCTCGGCTTTTGC, 4 and 5R AGGGACTAGGAGTGTCAGGATG, 4 and 5F CAAAGTGCTTGGTTCAGCCTC, 6aR GAAGGAGCTCCAGGCGGGGTTG, 6aF TAGGGGAGAAGAGGCACGTTG, 6bR GGATCACCTGAGGTCAGGAGTT, 6bF TTCACCGTGTTAGCCAGGA.

Capillary electrophoresis was performed using a 3130 automated sequencer (ABI Prism) and analysis using Gene Mapper software (ABI Prism). We used RefSeq: NCBI RefSeq: NG_009385.2 and NM_000451.3, NM_006883.2 for variation calling and found variants were described according to recommendations for the description of DNA changes (HGVS). The clinical significance of the variants was determined using genomic databases—ClinVar, Varsome, LOVD, and Ensembl. We double checked (two runs) the pathogenic variants. The origin of the variants was determined in the parents whenever possible. In cases of chromosomal aberrations, the status of the *SHOX* gene was always verified by MLPA and/or FISH. The significance of particular co-morbidities for the detection of *SHOX* variants was determined by Fisher’s exact test, and the probability of *SHOX* mutation occurrence with particular co-morbidities was calculated using a forward/stepwise logistic regression model. All calculations were performed by the analytical company Acrea using PS Imago Pro software.

*SHOX*-negative patients were further tested by CMA and targeted sequencing of relevant genes (e.g., rasopatias, *NPR2* etc.) consistently with their further symptoms.

## Results

We detected 27 (15.5%) mutations of *SHOX* in the short stature cohort and 15 (16.5%) mutations in the control cohort (*p* > 0.01) (Table 1).

**Table 1:** Frequency of the particular SHOX variants in short stature and control cohort.

	Methods (N)	SHOX mutations	45,X; 46, Xi(X)(q10); large del	%	Mosaic 45,X/46, XX or 46,XY —large del	%	Mosaic 45,X/47, XXX or 46,Xidic(Y) (del and dup)	%	Sequence variants	%	Large dup	%	Del of regulatory elements	%	Dup of regulatory elements	%	Total detected aberrations	%	F (*N* = 101)	M (*N* = 73)
Below -2SDS	karyotyping FISH MLPA	Aberration of sex chromosomes	3	1.7	2	1.2	3	1.7	0	0.0	0	0.0	0	0.0	0	0.0	8	4.6	6	2
	MLPA sequencing	LWD	4	2.3	0	0.0	0	0.0	0	0.0	0	0.0	1	0.6	0	0.0	5	2.9	4	1
		Nonsydromic short stature	0	0.0	0	0.0	0	0.0	3 (VUS)	1.7	1 (VUS)	0.6	0	0.0	10 (VUS)	5.7	14	8.0	9	5
	Total (*N* = 174)		7	4.0	2	1.2	3	1.7	3	1.7	1	0.6	1	0.6	10	5.7	27	15.5	19 (10.9%)	8 (4.6%)
Above -2SDS	Karyotyping FISH MLPA	Aberration of sex chromosomes	0	0.0	2	2.2	1	1.1	0	0.0	0	0.0	0	0.0	0	0.0	3	3.3	1	2
	MLPA sequencing	LWD	1	1.1	0	0.0	0	0.0	0	0.0	0	0.0	0	0.0	0	0.0	1	1.1	1	0
		Nonsyndromic	0	0.0	0	0.0	0	0.0	1 (VUS)	1.1	3 (VUS)	3.3	3(VUS)	3.3	4(VUS)	4.4	11	12.1	3	8
	Total (*N* = 91)		1	1.1	2	2.2	1	1.1	1	1.1	3	3.3	3	3.3	4	4.4	15	16.5	5 (5.5%)	10 (11.0%)
*N* (265)			8		4		4		4		4		4		14		42		156	109

**Figure 2:**
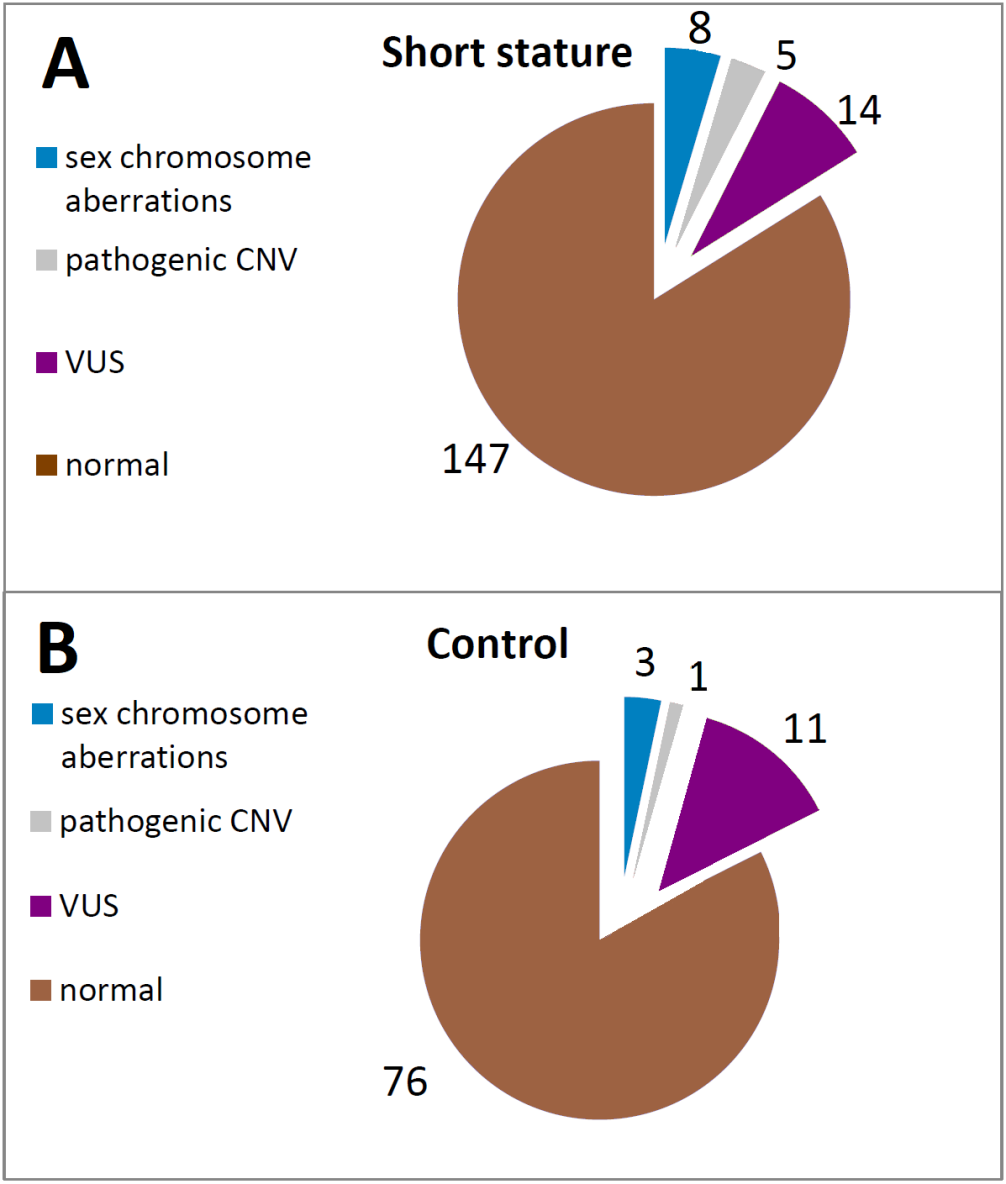
Frequency of the particular *SHOX* aberrations in individuals of the short stature (A) and control cohort (B).

**Figure 3:**
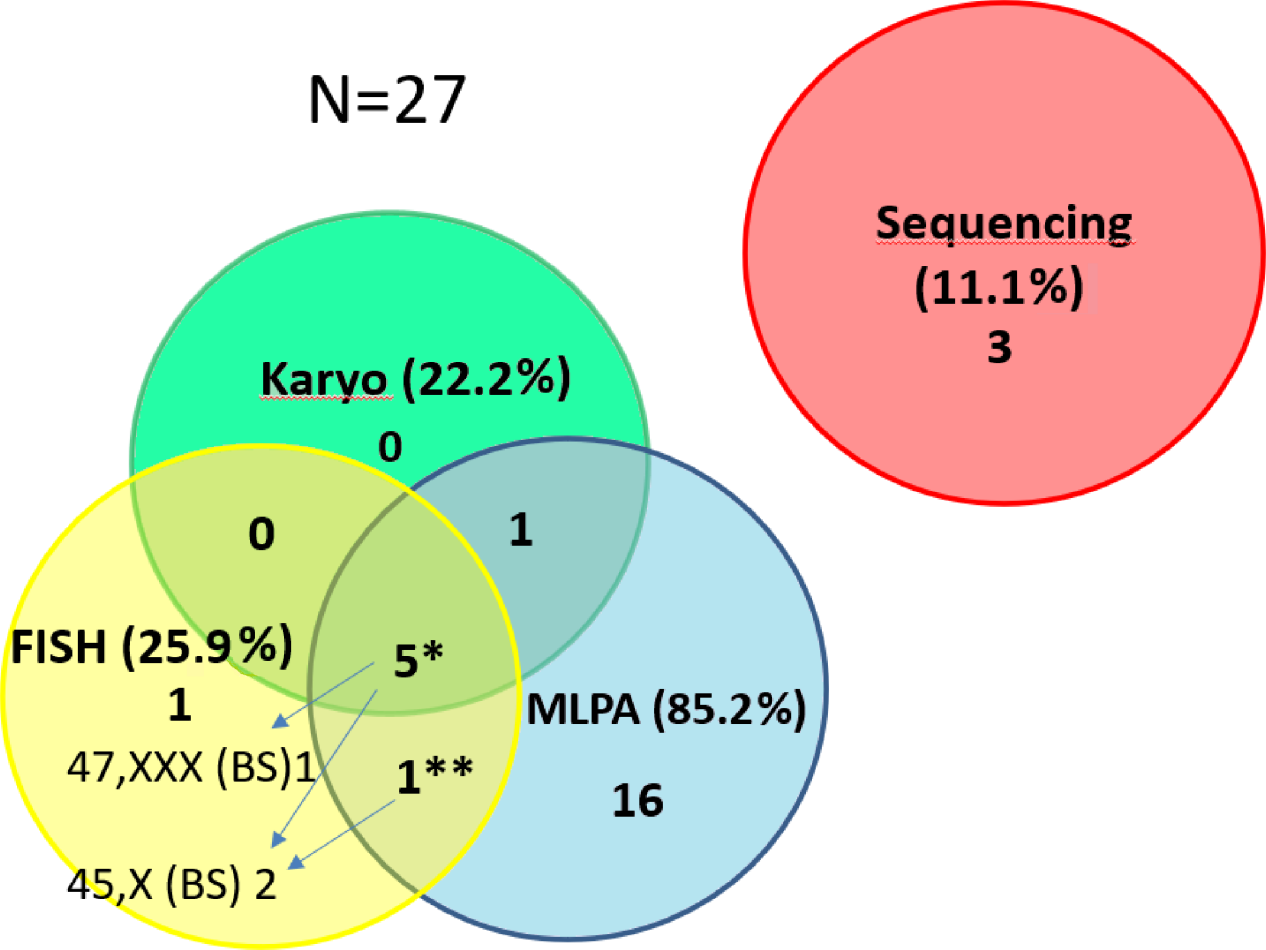
Contribution of the particular methods to the detection of *SHOX* variants in the short stature cohort. The number of detected CNV for the particular methods in the short stature cohort *N* = 27. The detection of pathogenic findings was made by three methods in 5 samples* and by two methods in one sample**. However, each method contributes differently to clarify patients’ phenotype. We were able to detect different clone by FISH on a buccal smear (47,XXX in 1 out of 5 samples or 45,X in 1 out of 5 samples). In one sample** we detected duplication of SHOX by MLPA but 45,X clone was discovered by FISH on the buccal smear. By FISH 1 case (mosaic of 45,X) was exclusively detected. The method helped to specify altogether the findings in 3 cases (arrows) and the result concordant with MLPA and karyotyping was achieved in further 3 cases by FISH. (The detection rate for the particular methods related to the number of performed tests is stated in the text).

The aberrations of *SHOX* caused by structural or numerical changes in sex-chromosomes were revealed by karyotyping in a total of 6 out of 169 (3.55%) patients with short stature (Fig. 2, Table S1). All the cases were also confirmed and specified by MLPA in our study (Fig. 3). Of these, we have also obtained concordant results in 3 cases with FISH on a different tissue (buccal smear). However, we also had discordant findings on the basis of FISH performed on a buccal swab. We could discover the presence of chromosomally different cell clones—45,X or 47,XXX—which were not detected by karyotyping in 4 cases overall in the short stature group using FISH on buccal smears—see cases below (Table S1). The overall detection rate using FISH is higher than karyotyping—7 out of 52 (13.46%) in short stature patients (Fig. 3).

Case 1 (Table S1): Small mosaic 47,XXX (9%) was found by FISH in buccal swab of girl (ID 1255/15) manifesting the phenotype of TS with karyotype 45,X from peripheral blood. The patient was a mosaic of TS.

**Figure 4:**
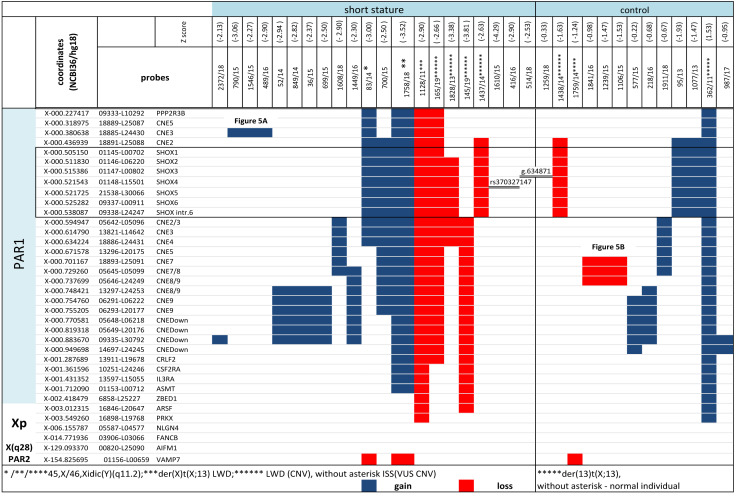
CNV detected by MLPA and Sanger sequencing in the short stature and control cohort. CNV detected by MLPA and Sanger sequencing in the short stature and control cohort. Diagnosis in the patients is marked with an asterisk, ISS patients are without an asterisk.

Case 2 (Table S1): A normal male karyotype was detected in a patient (ID 1758/18) with short stature (-3.52 Z-score) and IUGR. Through MLPA we discovered *SHOX* duplication, which finally appeared to be idic(Y) by FISH. Moreover, we confirmed the presence of minor clone 45,X in the patient’s buccal swab. We had also verified the presence of such a cell clone through karyotyping 45,X[3]/46,X,idic(Y)(q11.223)[35] by subsequent checking of additional specimens. This mosaic and idic(Y) would not be detected without FISH and MLPA, respectively. The case was further delineated by CMA (Table S1, Fig. 4). The conclusion of the investigation was mosaic of monosomy X and idic(Y), which could better explain the short stature in the patient.

Case 3 (Table S1): Large deletion of Yq was suspected on the basis of karyotyping (Table S1) in a boy with an unexplained growth restriction (-3.0 Z-score) (ID 83/14) and IUGR. *SHOX* duplication was detected by MLPA. The finding was concluded as idic(Y) based on the results of MLPA and FISH with *SHOX* probe. A minor clone of 45,X was also detected in the buccal smear of the patient by FISH (Table S1) which could explain growth restriction in the patient.

Case 4 (Table S1): The minor mosaic 45,X (13%) would not have been detected in the asymptomatic female with short stature (-3.31 Z-score) (ID 79/19), normal female karyotype and normal MLPA, if we had not performed FISH on her buccal smear. We subsequently found that a mosaic 45,X[6]/46,XX[6] and mosaic 45,X[24]/46,XX[84] were recorded as prenatal karyotypes in the female from her amniotic fluid and cord blood, respectively, but these were not detected in peripheral lymphocytes postnatally. We assume that X-aneuploidy cells were suppressed from mitotic division during development in at least some tissues.

CNV of *SHOX* were discovered in an additional 16 individuals with normal karyotypes and normal FISH results (Figs. 3 and 4). Out of these 4 pathogenic deletions spanning the whole *SHOX* gene and 1 pathogenic deletion of regulatory elements of *SHOX* (Table 1, Fig. 4). In one case deletion resulted from balanced translocation t(X;13) in the mother. CNV detected in a further 11 patients were classified as VUS (Fig. 2). Overall detection rate of MLPA was 13.2% (23 out of 174 tested). The duplications of *SHOX* regulatory elements were the most frequent mutation detected in the short stature cohort (Table 1). The frequency of sequence variants in the coding regions and flanking areas of these regions by Sanger sequencing was 1.95% (3 out of 154 tested) (Fig. 3). However, only intronic VUS variants were detected—heterozygous nucleotide change NM_000451.3 (*SHOX*): c.545-10T>C (rs370327147) in the 4th intron NM_000451.3 (*SHOX*): c.486+45G>A in the 3rd intron both inherited (Fig. 4). The same spectrum of *SHOX* aberrations has also been found in the control cohort (Figs. 2 and 3, Table 1). Despite the fact that we detected a higher rate of *SHOX* variants in females than in males in the short stature group, there was no significant difference between frequencies of variants in both genders. However, a conversed ratio in *SHOX* variant frequencies between females and males was detected in the control group compared to the short stature group (Table 1). *SHOX* variants in the male control cohort were more than twice as high as in males of the short stature group (*p* = 0.0519; *p* < 0.10).

Short stature as a stand-alone marker was insignificant (*p* = 0.8621; *p* > 0.01) for the detection of *SHOX* variants, either pathogenic or VUS, using Fisher’s exact test (Table 2).

**Table 2:** Assesment of particular characteristics significance for detection of SHOX mutations.

**Clinical feature**	**N**	**Total without variants (%)**	**SHOX patogenic mutations *N* = 6 (%)**	***P* value**	**Sex chromosome aberrations *N* = 11 (%)**	***P* value**	**SHOX VUS *N* = 25 (%)**	***P* value**
**height (−2 SDS)**	265	147 (65.9)	5 (83.3)	ns	8 (72.7)	ns	14 (56.0)	ns
**weight <3. percercentile**	209	46 (27.5)	2 (33.3)	ns	4 (36.4)	ns	2 (8.0)	ns
**BMI >90. percercentile**	209	6 (3.6)	0 (0.0)	ns	2 (18.2)	ns	4 (16.0)	0.0276 (*p* < 0.05)
skeletal markers^*^	216	22 (12.6)	5 (83.3)	0.0003 (*p* < 0.01)	4 (36.4)	ns	6 (22.2)	ns
**Madelung deformity**	216	2 (1.2)	4 (66.7)	0.00001 (*p* < 0.01)	2 (18.2)	0.0181 (*p* < 0.05)	0 (0.0)	ns
**heart malformations**	216	16 (9.2)	0 (0.0)	ns	1 (9.1)	ns	0 (0.0)	ns
**other congenital malformations**	216	2 (1.2)	0 (0.0)	ns	1 (9.1)	ns	2 (7.4)	ns
**microcephaly/macrocephaly**	216	29 (16.7)	0 (0.0)	ns	0 (0.0)	ns	1 (3.7)	ns
**facial dysmorphia/abnormality of ears**	216	23(13.2)	1 (16.7)	ns	3 (27.3)	ns	3 (11.1)	ns
**micromandible/high arched palate/cleft palate**	216	4 (2.3)	0 (0.0)	ns	1 (9.1)	ns	2 (7.4)	ns
**hypospadia/hypogonadism**	216	0 (0.0)	0 (0.0)	ns	2 (18.2)	0.0032 (*p* < 0.01)	0 (0.0)	ns
**neurodevelopmental impairment**	216	25 (14.5)	0 (0.0)	ns	1 (9.1)	ns	5 (18.5)	ns
**familial growth restriction**	216	56 (32.6)	3 (50.0)	ns	0 (0.0)	ns	8 (29.6)	ns
**intrauterine growth restriction (IUGR)**	208	42 (25.3)	0 (0.0)	ns	5 (45.5)	ns	3 (11.1)	ns

**Notes.**

* disproportionate growth, mesomelia, rhizomelia, wrist changes, shortening of the fourth and fifth metacarpal bones, tibial bowing, muscular hypertrophy

MD Madelung deformity CM congenital malformations

The typical skeletal signs of LWD, including Madelung deformity and disproportionate growth, positively correlate with the findings of pathogenic CNV *SHOX* variants (Table 2) by Fisher’s exact test. However, we did not detect the same correlation in the group of patients with detected VUS variants (Table 2). The correlation with increased BMI was observed in this subgroup (Table 2). Hypospadia, hypogonadism and Madelung deformity showed increased significance in our study due to sex chromosome aberrations (Table 2). The positive predictive value of the screening (karyotyping, MLPA, FISH) was low (15.5%) if we depend only on short stature defined as deviation larger than -2SDS. *SHOX* variants—especially VUS variants involving duplications and intronic variants in *SHOX* and duplications and deletions of enhancers—were also recorded in children with smaller deviations (Table S1). A broad range of phenotypes from variable non-specific symptoms to asymptomatic accompanied these variants. Further scored co-morbidities and characteristics were insignificant for the detection of *SHOX* variants (Table 2).

Short stature increases the likelihood ratio for the detection of relevant *SHOX* aberrations leading to determination of the diagnosis, provided it is accompanied with disproportionate growth or markers of skeletal dysplasia or Madelung deformity in patients by logit regression (*p* = 0.000 and 0.011).

## Discussion

**Figure 5:**
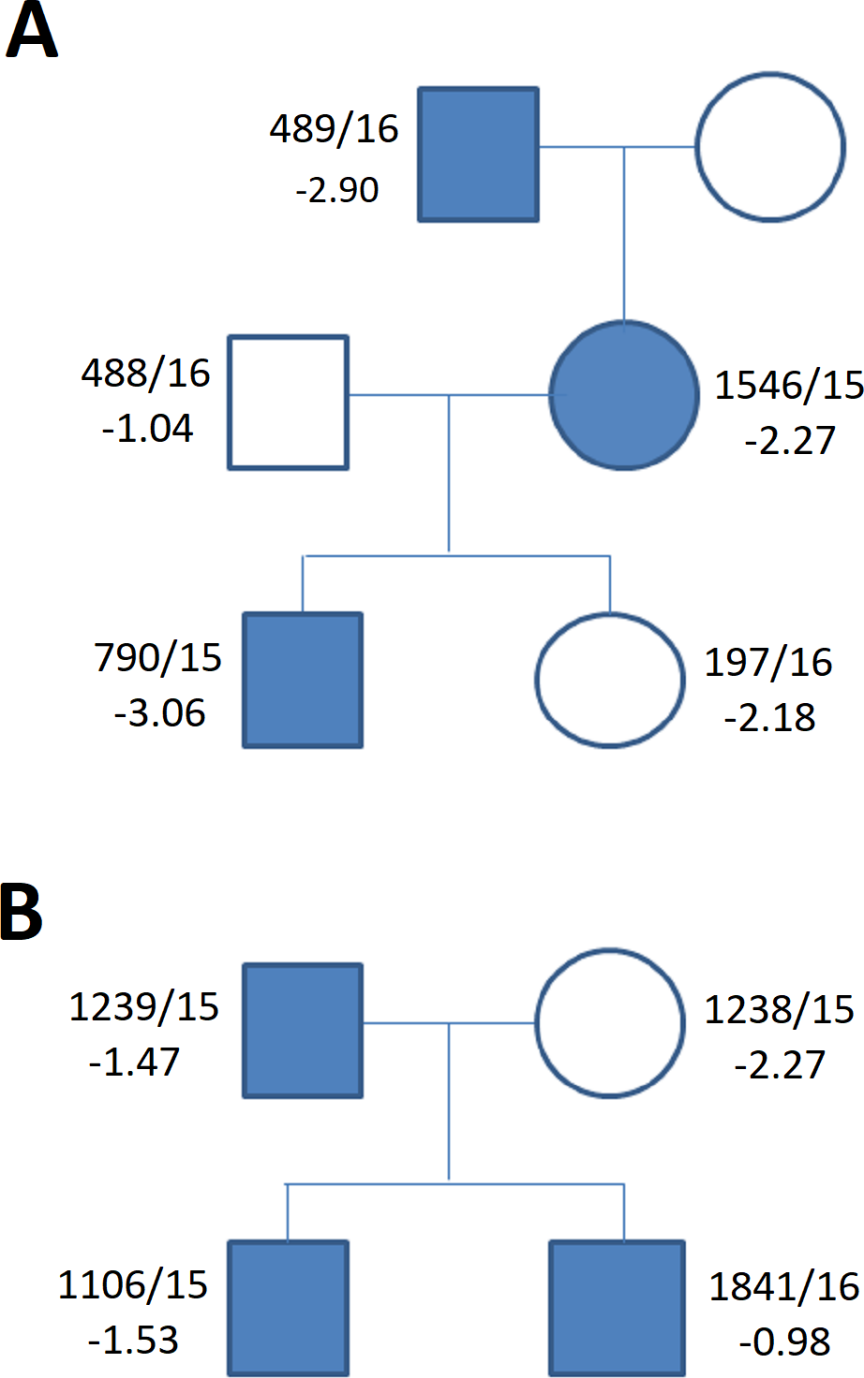
Family tree in (A) duplication of the upstream enhancers elements (B) deletion of the downstream enhancers elements. The family tree in (A) duplication of the single upstream enhancer element CNE3 in propositus with ISS and cleft palate (Table 1, Table S1) (B) recurrent deletion of the downstream enhancers elements - 47.5 kb CNE7-CNE9 in individuals of the control group (Table 1, Table S1) (blue symbols—detected variant).

Screening for *SHOX* variants in the short stature group yielded 15.5% of the variants generally in our study. It shows that short stature as a stand-alone indicator for *SHOX* abnormalities has a low positive predictive value. Only VUS variants were detected in the nonsyndromic short stature group. The correlation of short stature with findings of *SHOX* aberrations was not significant in any of the particular variation subgroups by Fisher’s exact test if we calculate -2SDS as a cut-off value. However, the small size of the particular subgroups (with detected variants) means that the conclusion might be biased. Despite the fact that we were not able to prove significant difference between short stature and control groups it was evident that heterozygous loss of *SHOX* (large deletions) consistent with LWD and TS diagnosis in patients has an impact on the linear growth of patients whereas another variants showed milder impact. Moreover, we observed conversed ratio in the frequencies of *SHOX* variants in females and males when we compare the short stature group and the control group. A higher frequency of *SHOX* mutations was reported in the short stature females than in males (Rappold et al., 2007). As we detected a higher rate of *SHOX* variants in males of the control cohort than in the short stature group we assume that the impact of these variants on the height of females might be more pronounced than in males. The adverse effect of estrogen at the *SHOX* deficient growth plate in LWD females has been previously hypothesized (Fukami et al., 2004). It is questionable whether males with *SHOX* variants have an advantage against females in achieved final height because of later maturation. More study is necessary to prove the hypothesis. Disproportionate growth/other skeletal markers for LWD (*p* < 0.01) and Madelung deformity (*p* < 0.01) were significant features for the relevant *SHOX* CNV aberrations, which has been described previously (Wolters et al., 2013; Dávid et al., 2017; Rappold et al., 2007; Zapletalova et al., 2010). The likelihood ratio for *SHOX* CNV aberrations increases when Madelung deformities and/or disproportionate stature/skeletal markers for LWD manifest in patients. These markers reflected the haploinsufficiency of *SHOX* in case of the complete deletion without short stature in the mother of the boy with short stature (Table S1—cases 1437, 1438/14). On the contrary we have not confirmed the significant correlation between sex chromosome aberrations and skeletal markers except for Madelung deformity which might support the conclusions of the study written by Soucek et al. (2013). Significantly higher BMI and correlation with dysmorphic features have been observed in some studies (Dávid et al., 2017; Rappold et al., 2007; D’angelo et al., 2018). We observed the correlation of higher BMI in the group of patients with VUS variants but we did not prove correlation with skeletal markers in this group. However, we should take into account that the mean of age in our short cohort was 8.6 years so probably the majority of patients did not manifest skeletal deformities at the time. Hypospadia, hypogonadism, and ambiguous genitalia are more obligate sign for mosaics 45,X/46,XY or 45,X/46,Xidic(Y)(q11.2) than short stature (Hes et al., 2009; Kaprova-Pleskacova et al., 2013; Nomura et al., 2015). Thus we detected significant correlation in patient with aberration of sex chromosomes (*p* < 0.01) but it was not detected in patients with CNV variants of *SHOX*. Hypospadia was detected in a single case—in a male without short stature with mosaic 45,X/46,XY in peripheral lymphocytes, buccal smear and tissue from testicle biopsy, but did not manifest in cases with minor mosaic 45,X and idic(Y) and short stature in our study. We also assessed additional characteristics but without significant results. This study was performed to assess short stature as a predictive marker for *SHOX* aberrations and to assess the contribution of screening (consists of karyotyping, MLPA, sequencing and FISH methods) to the detection of *SHOX* abnormalities. The aneuploidies of sex-chromosomes (including TS) were also included in this study as mosaic of monosomy X might be hidden among patients with isolated short stature. Mosaics of TS might manifest with isolated short stature in early childhood and the correct diagnosis might not be made until reproductive age. However, the treatment of GH should be administered before pubertal initiation (Shankar & Backeljauw, 2018). Phenotypic data were also compared for children with TS in whom haploinsufficiency of *SHOX* is thought to be responsible for the height deficit as targeted exclusion of both syndromes (TS and LWD) is usually performed in children with growth restriction below the 3rd percentile (Shankar & Backeljauw, 2018). Despite the opinion that FISH on different tissue as a routine in short stature girls is not necessary (Shankar & Backeljauw, 2018) we found that occult mosaicism might be a factor that may influence the growth of individuals. A great number of cases of TS seem to be mosaic (Hook & Warburton, 2014). Some of the mosaics might be missed, if we investigate a limited number of cells and only one tissue. Moreover, some studies showed that the number of disomic (XX or XY) cells increases during the lifetime which might complicate detection (see cases in the result section) of cryptic monosomic clones (Denes et al., 2015). The discovered cryptic mosaic of normal cell clones or cell clones with extra copies of X chromosomes might explain the mechanism through which seemingly monosomic pregnancies are rescued from adverse outcomes (Hook & Warburton, 2014). Male with mosaic 45,X/46,XY karyotypes with isolated short stature and otherwise normal male phenotype have been also reported (Richter-Unruh et al., 2004). The prevalence of this disorder in cohort of ISS children is not known (Dauber, Rosenfeld & Hirschhorn, 2014). We assume that investigating minor mosaics in different tissues might bring to light new aspects of unexplained isolated growth restrictions. We were able to explain short stature in at least 3 (out of 174) cases where the initially normal finding or found variants (by MLPA or karyotyping) were not directly associated with short stature (duplication of *SHOX*). People with triple *SHOX* due to sex chromosome trisomy usually show tall stature (Ogata et al., 2000; Upners et al., 2017). However, this is not always rule in individuals with microduplications of PAR1 involving *SHOX* or its regulatory sequences (Benito-Sanz et al., 2011; Valetto et al., 2016; Thomas et al., 2009; Roos, Nielsen & Tümer, 2009). We would have attributed short stature to duplication of *SHOX* resulting from idic(Y) if we had not performed FISH on buccal swabs to detect cryptic 45,X clones. On the contrary, in the cases where a mosaic of 45,X/46,X,idic(Y)(q11.22) was revealed in the blood and no monosomic cells were detected in buccal smears, we could assume that cells with an extra copy of *SHOX* are present in the growth plates of chondrocytes, which compensate the heterozygous loss in some cells in individuals without short stature (control 1759/14) (Fig. 4, Table S1). It implies that an explanation might be achieved for normal growth in individuals, at least. However, phenotypes of patients might be variable and probably reflect the distribution of monosomic cells (Hes et al., 2009; Kaprova-Pleskacova et al., 2013). We have observed that only the small 56 kb duplication of upstream regulatory elements segregated with proportionate short stature (Fig. 5). On the contrary, duplication of downstream regulatory elements showed variable patterns of segregation, including tall stature. The significance of such a finding has been commented by several authors (Hirschfeldova & Solc, 2017; Benito-Sanz et al., 2011). Duplication of *SHOX* and its enhancers may represent one of the susceptibility factors influencing human height (Roos, Nielsen & Tümer, 2009). We have also observed that the deletion of *SHOX* enhancers did not always segregate with short stature and LWD phenotype. This was especially pronounced in a family with recurrent 47.5 kb deletion in the *SHOX* downstream region detected in male members of the family in the control cohort (SDS less than -2SDS). The deletion of SHOX enhancers was located on the Y chromosome (Fig. 5). Because of the frequent recombination between both PAR1 regions, it is sometimes difficult to determine the origin of such a mutation. But we confirmed in the family that deletion was paternally inherited on the Y chromosome. Variable impact of the enhancers deletion to the height of the individuals even within families was described previously (Kant et al., 2013). All male family members with the variant were without features suggesting LWD at the time of investigation. This deletion of enhancers was the only one where we confirmed its transmission on the chromosome Y. It would be interesting to research whether there is difference in the phenotype of the patients, dependent on the type of sex chromosome carrying the CNV. MLPA and FISH showed the highest detection rate of *SHOX* aberration. FISH is a convenient method for detection of mosaics in different tissue than peripheral blood. It is a complementary method to MLPA or karyotyping for detection of mosaic X monosomy. The detection rate of sequencing is the lowest compared to the other method in patients with only short stature, which raises the question of whether sequencing is meaningful especially in patients with short stature without any other clinical symptoms. We detected only intronic VUS variants which are not causal for LWD but we suppose they might modify the linear growth. Among *SHOX*-negative patients we have further discovered 1 patient with duplication 17q12(34,822,465-36,283,612)x3 by CMA and mutations causing Cornelia de Lang syndrome (1), Noonan syndrome (2), cardiofaciocutaneous syndrome (1), Legius syndrome (1), hypophosphatemic rachitis (1) and mutation in *ANKRD* 11(1) in the short stature cohort so far.

## Conclusions

Despite the fact that we could not prove that there is a significant correlation between *SHOX* variants and growth restriction using Fisher’s exact test, the logit regression implies that short stature is a significant predictor for the risk of pathogenic *SHOX* variants, provided it is accompanied with either Madelung deformity or disproportionate stature/other skeletal signs for LWD. This fact is indicative that a very careful physical examination, including measurement of body proportions, is essential before testing and it increases the effectiveness of the testing by discovering of the relevant *SHOX* variants leading to unambiguous diagnosis. The majority of the variants detected in the short stature cohort were classified as VUS and we assume that they only modify the growth of individuals. Most of them were inherited and very often from parents with normal height. These cases are inconclusive. On the other hand, the result of our extensive screening showed us that cryptic or tissue mosaics of monosomy X might be missed if we do not test children with no obvious markers of *SHOX* deficiency. *SHOX* variants in the male control cohort were more than twice as abundant than in males of the short stature group. In females the frequency of *SHOX* variants was higher in the short stature group than in the control group. It might imply that the impact of *SHOX* variants on height may be more pronounced in females. However, further study should be carried out. We assume the influence of other genes that cooperate in the process of growth and differentiation of chondrocytes together with another internal factors (hormonal etc.) including the time differences in maturation of both genders. MLPA is reliable as a frontline method for the detection of *SHOX* mutations including sex chromosome aberrations. Moreover, it is more convenient than time-consuming karyotyping for the fast targeted exclusion of *SHOX* variants including sex chromosome aberrations in screening of large groups of short stature patients. However, the detection of mosaic of monosomy X is limited by the proportion of aberrant clones. We also suggest adding FISH on different tissue than peripheral blood to verify sex-chromosome constitution, especially in cases with karyotypes: 45,X; mosaic 45,X/46,XX or 46,XY; 46,Xidic(Y) detected from blood; in children, where mosaic 45,X was detected prenatally but was not confirmed from peripheral blood.

##  Supplemental Information

10.7717/peerj.10236/supp-1 Table S1 Patients with SHOX variants *cut off for blood F (X0) - 7.43 %, F(XXX)-2.0 % ; M (X0) - 7.5 %, M(XXY) - 4.9 %;buccal smear F(X0)- 9,0 % , F(XXX) - 4.5 %;M(X0)- 3.5 %, M(XXY)-5.9% (ISS- idiopathic short stature;MD-Madelung deformity;NA not analysed)

10.7717/peerj.10236/supp-2 Supplemental Information 2 Statistic calculations SS, short stature

## Additional Information and Declarations

### Data Availability

The following information was supplied regarding data availability: All raw data are available in the Supplemental Files.
