# Supplementary material for: Short stature and SHOX (Short stature homeobox) variants—efficacy of screening using various strategies
Source: PeerJ. 2020 Nov 17;8:e10236. doi: 10.7717/peerj.10236 (PMC7678493; doi:10.7717/peerj.10236)
Supplement: Supplemental Information 2 — SS, short stature [file peerj-08-10236-s002.zip › Statistics/logistic regression.docx]

LOGISTIC REGRESSION VARIABLES SHOX_overall

/METHOD=BSTEP(COND) RR1normal 0 Skeletal markers LWD MD

/SAVE=PRED

/CRITERIA=PIN(0.05) POUT(0.10) ITERATE(30) CUT(0.17).

**Logistic Regression**

| **Case Processing Summary** | | | |
| --- | --- | --- | --- |
| Unweighted Cases^a^ | | N | Percent |
| Selected Cases | Included in Analysis | 216 | 81,5 |
|  | Missing Cases | 49 | 18,5 |
|  | Total | 265 | 100,0 |
| Unselected Cases | | 0 | ,0 |
| Total | | 265 | 100,0 |

| a. If weight is in effect, see classification table for the total number of cases. |
| --- |

| **Dependent Variable Encoding** | |
| --- | --- |
| Original Value | Internal Value |
| 0 | 0 |
| 1 | 1 |

**Block 0: Beginning Block**

| **Classification Table^a,b^** | | | | | |
| --- | --- | --- | --- | --- | --- |
|  | Observed | | Predicted | | |
|  |  |  | SHOX_celkem | | Percentage Correct |
|  |  |  | 0 | 1 |  |
| Step 0 | SHOX_total | 0 | 0 | 179 | ,0 |
|  |  | 1 | 0 | 37 | 100,0 |
|  | Overall Percentage | |  |  | 17,1 |

| a. Constant is included in the model. |
| --- |
| b. The cut value is ,170 |

| **Variables in the Equation** | | | | | | | |
| --- | --- | --- | --- | --- | --- | --- | --- |
|  | | B | S.E. | Wald | df | Sig. | Exp(B) |
| Step 0 | Constant | -1,576 | ,181 | 76,203 | 1 | ,000 | ,207 |

| **Variables not in the Equation** | | | | | |
| --- | --- | --- | --- | --- | --- |
|  | | | Score | df | Sig. |
| Step 0 | Variables | SS(1), normal (0) | 2,988 | 1 | ,084 |
|  |  | Skeletal markers LWD | 20,263 | 1 | ,000 |
|  |  | MD | 19,600 | 1 | ,000 |
|  | Overall Statistics | | 35,719 | 3 | ,000 |

**Block 1: Method = Backward Stepwise (Conditional)**

| **Omnibus Tests of Model Coefficients** | | | | |
| --- | --- | --- | --- | --- |
|  | | Chi-square | df | Sig. |
| Step 1 | Step | 29,368 | 3 | ,000 |
|  | Block | 29,368 | 3 | ,000 |
|  | Model | 29,368 | 3 | ,000 |

| **Model Summary** | | | |
| --- | --- | --- | --- |
| Step | -2 Log likelihood | Cox & Snell R Square | Nagelkerke R Square |
| 1 | 168,460^a^ | ,127 | ,212 |

| a. Estimation terminated at iteration number 5 because parameter estimates changed by less than ,001. |
| --- |

| **Classification Table^a^** | | | | | |
| --- | --- | --- | --- | --- | --- |
|  | Observed | | Predicted | | |
|  |  |  | SHOX_celkem | | Percentage Correct |
|  |  |  | 0 | 1 |  |
| Step 1 | SHOX_overall | 0 | 123 | 56 | 68,7 |
|  |  | 1 | 11 | 26 | 70,3 |
|  | Overall Percentage | |  |  | 69,0 |

| a. The cut value is ,170 |
| --- |

| **Variables in the Equation** | | | | | | |
| --- | --- | --- | --- | --- | --- | --- |
|  | | B | S.E. | Wald | df | Sig. |
| Step 1^a^ | SS(1), normal(0) | -1,064 | ,443 | 5,766 | 1 | ,016 |
|  | Skeletal markers LWD | 1,595 | ,448 | 12,644 | 1 | ,000 |
|  | MD | 2,321 | ,916 | 6,417 | 1 | ,011 |
|  | Constant | -1,327 | ,358 | 13,730 | 1 | ,000 |

| **Variables in the Equation** | | |
| --- | --- | --- |
|  | | Exp(B) |
| Step 1^a^ | SS(1), normal (0) | ,345 |
|  | Skeletal markers LWD | 4,926 |
|  | MD | 10,182 |
|  | Constant | ,265 |

| a. Variable(s) entered on step 1: RR(1), normální (0), Skeletal markers LWD, MD. |
| --- |

| **Model if Term Removed^a^** | | | | | |
| --- | --- | --- | --- | --- | --- |
| Variable | | Model Log Likelihood | Change in -2 Log Likelihood | df | Sig. of the Change |
| Step 1 | SS(1), normal(0) | -87,074 | 5,688 | 1 | ,017 |
|  | Skeletal markers LWD | -90,436 | 12,412 | 1 | ,000 |
|  | MD | -87,968 | 7,475 | 1 | ,006 |

| a. Based on conditional parameter estimates |
| --- |

ROC PRE_1 BY SHOX_celkem (1)

/PLOT=CURVE(REFERENCE)

/CRITERIA=CUTOFF(INCLUDE) TESTPOS(LARGE) DISTRIBUTION(FREE) CI(95)

/MISSING=EXCLUDE.

**ROC Curve**

| **Case Processing Summary** | |
| --- | --- |
| SHOX_overall | Valid N (listwise) |
| Positive^a^ | 37 |
| Negative | 179 |
| Missing | 49 |

| Larger values of the test result variable(s) indicate stronger evidence for a positive actual state. |
| --- |
| a. The positive actual state is 1. |


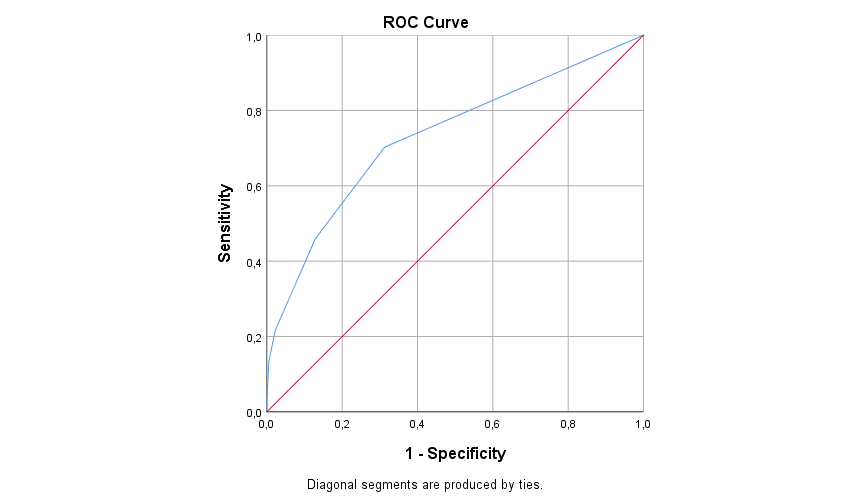


| **Area Under the Curve** |
| --- |
| Test Result Variable(s): Predicted probability |
| Area |
| ,731 |

| The test result variable(s): Predicted probability has at least one tie between the positive actual state group and the negative actual state group. Statistics may be biased. |
| --- |
