# Supplementary material for: Short stature and SHOX (Short stature homeobox) variants—efficacy of screening using various strategies
Source: PeerJ. 2020 Nov 17;8:e10236. doi: 10.7717/peerj.10236 (PMC7678493; doi:10.7717/peerj.10236)
Supplement: Supplemental Information 2 — SS, short stature [file peerj-08-10236-s002.zip › Statistics/PPV.docx]

CROSSTABS

/TABLES=SS1normal0 BY SHOX_celkem

/FORMAT=AVALUE TABLES

/STATISTICS=CHISQ

/CELLS=COUNT ROW

/COUNT ROUND CELL.

**Crosstabs**

| **Case Processing Summary** | | | | | | |
| --- | --- | --- | --- | --- | --- | --- |
|  | Cases | | | | | |
|  | Valid | | Missing | | Total | |
|  | N | Percent | N | Percent | N | Percent |
| SS(1), normal (0) * SHOX_total | 265 | 100,0% | 0 | 0,0% | 265 | 100,0% |

| **SS(1), normal (0) * SHOX_overall Crosstabulation** | | | | | |
| --- | --- | --- | --- | --- | --- |
|  | | | SHOX_total | | Total |
|  |  |  | 0 | 1 |  |
| Count | SS(1), normal (0) | 0 | 75 | 16 | 91 |
|  |  | 1 | 146 | 28 | 174 |
|  | Total | | 221 | 44 | 265 |
| % within SS(1), normal (0) | SS(1), normal (0) | 0 | 82,4% | 17,6% | 100,0% |
|  |  | 1 | 83,9% | 16,1% | 100,0% |
|  | Total | | 83,4% | 16,6% | 100,0% |

| **Chi-Square Tests** | | | | | |
| --- | --- | --- | --- | --- | --- |
|  | Value | df | Asymptotic Significance (2-sided) | Exact Sig. (2-sided) | Exact Sig. (1-sided) |
| Pearson Chi-Square | ,096^a^ | 1 | ,757 |  |  |
| Continuity Correction^b^ | ,018 | 1 | ,892 |  |  |
| Likelihood Ratio | ,095 | 1 | ,758 |  |  |
| Fisher's Exact Test |  |  |  | ,862 | ,441 |
| Linear-by-Linear Association | ,095 | 1 | ,757 |  |  |
| N of Valid Cases | 265 |  |  |  |  |

| a. 0 cells (,0%) have expected count less than 5. The minimum expected count is 15,11. |
| --- |
| b. Computed only for a 2x2 table |

CROSSTABS

/TABLES=SS1normal0 BY delSHOXX45X46XiXqdeletionSHOX

mosaic45X46XXor46XYdeletionSHOX mosaic45X47XXXor46XidicYdeletionandduplicationSHOX mutaceSHOXX

dupSHOXX delregulSHOXX delregulSHOXY dupregulSHOX

/FORMAT=AVALUE TABLES

/STATISTICS=CHISQ

/CELLS=COUNT ROW

/COUNT ROUND CELL.

**Crosstabs**

| **Case Processing Summary** | | | | | | |
| --- | --- | --- | --- | --- | --- | --- |
|  | Cases | | | | | |
|  | Valid | | Missing | | Total | |
|  | N | Percent | N | Percent | N | Percent |
| SS(1), normal(0) * del SHOX (X); 45,X; 46,Xi(Xq);(deletion SHOX) | 265 | 100,0% | 0 | 0,0% | 265 | 100,0% |
| SS(1), normal (0) * mosaic 45,X/46,XX or 46,XY (deletion SHOX) | 265 | 100,0% | 0 | 0,0% | 265 | 100,0% |
| SS(1), normal(0) * mosaic 45,X/47,XXX or 46,Xidic(Y)(deletion and duplication SHOX) | 265 | 100,0% | 0 | 0,0% | 265 | 100,0% |
| SS(1), normal(0) * mutace SHOX(X) | 265 | 100,0% | 0 | 0,0% | 265 | 100,0% |
| SS(1), normal(0) * dup SHOX(X) | 265 | 100,0% | 0 | 0,0% | 265 | 100,0% |
| SS(1), normal(0) * del regul SHOX (X) | 265 | 100,0% | 0 | 0,0% | 265 | 100,0% |
| SS(1), normal(0) * del regul SHOX (Y) | 265 | 100,0% | 0 | 0,0% | 265 | 100,0% |
| SS(1), normal (0) * dup regul SHOX | 265 | 100,0% | 0 | 0,0% | 265 | 100,0% |

**SS(1), normal (0) * del SHOX (X); 45,X; 46,Xi(Xq);(deletion SHOX)**

| **Crosstab** | | | | | |
| --- | --- | --- | --- | --- | --- |
|  | | | del SHOX (X); 45,X; 46,Xi(Xq);(deletion SHOX) | | Total |
|  |  |  | 0 | 1 |  |
| Count | SS(1), normal(0) | 0 | 90 | 1 | 91 |
|  |  | 1 | 167 | 7 | 174 |
|  | Total | | 257 | 8 | 265 |
| % within SS(1), normal(0) | SS(1), normal (0) | 0 | 98,9% | 1,1% | 100,0% |
|  |  | 1 | 96,0% | 4,0% | 100,0% |
|  | Total | | 97,0% | 3,0% | 100,0% |

| **Chi-Square Tests** | | | | | |
| --- | --- | --- | --- | --- | --- |
|  | Value | df | Asymptotic Significance (2-sided) | Exact Sig. (2-sided) | Exact Sig. (1-sided) |
| Pearson Chi-Square | 1,745^a^ | 1 | ,187 |  |  |
| Continuity Correction^b^ | ,889 | 1 | ,346 |  |  |
| Likelihood Ratio | 2,051 | 1 | ,152 |  |  |
| Fisher's Exact Test |  |  |  | ,270 | ,175 |
| Linear-by-Linear Association | 1,738 | 1 | ,187 |  |  |
| N of Valid Cases | 265 |  |  |  |  |

| a. 1 cells (25,0%) have expected count less than 5. The minimum expected count is 2,75. |
| --- |
| b. Computed only for a 2x2 table |

**SS(1), normal (0) * mosaic 45,X/46,XX or 46,XY (deletion SHOX)**

| **Crosstab** | | | | | |
| --- | --- | --- | --- | --- | --- |
|  | | | mosaic 45,X/46,XX or 46,XY (deletion SHOX) | | Total |
|  |  |  | 0 | 1 |  |
| Count | SS(1), normal(0) | 0 | 89 | 2 | 91 |
|  |  | 1 | 172 | 2 | 174 |
|  | Total | | 261 | 4 | 265 |
| % within SS(1), normální (0) | SS(1), normal (0) | 0 | 97,8% | 2,2% | 100,0% |
|  |  | 1 | 98,9% | 1,1% | 100,0% |
|  | Total | | 98,5% | 1,5% | 100,0% |

| **Chi-Square Tests** | | | | | |
| --- | --- | --- | --- | --- | --- |
|  | Value | df | Asymptotic Significance (2-sided) | Exact Sig. (2-sided) | Exact Sig. (1-sided) |
| Pearson Chi-Square | ,442^a^ | 1 | ,506 |  |  |
| Continuity Correction^b^ | ,018 | 1 | ,893 |  |  |
| Likelihood Ratio | ,420 | 1 | ,517 |  |  |
| Fisher's Exact Test |  |  |  | ,609 | ,425 |
| Linear-by-Linear Association | ,440 | 1 | ,507 |  |  |
| N of Valid Cases | 265 |  |  |  |  |

| a. 2 cells (50,0%) have expected count less than 5. The minimum expected count is 1,37. |
| --- |
| b. Computed only for a 2x2 table |

**SS(1), normal (0) * mosaic 45,X/47,XXX or 46,Xidic(Y)(deletion and duplication SHOX)**

| **Crosstab** | | | | | |
| --- | --- | --- | --- | --- | --- |
|  | | | mosaic 45,X/47,XXX or 46,Xidic(Y)(deletion and duplication SHOX) | | Total |
|  |  |  | 0 | 1 |  |
| Count | SS(1), normal(0) | 0 | 90 | 1 | 91 |
|  |  | 1 | 171 | 3 | 174 |
|  | Total | | 261 | 4 | 265 |
| % within SS(1), normal(0) | SS(1), normal (0) | 0 | 98,9% | 1,1% | 100,0% |
|  |  | 1 | 98,3% | 1,7% | 100,0% |
|  | Total | | 98,5% | 1,5% | 100,0% |

| **Chi-Square Tests** | | | | | |
| --- | --- | --- | --- | --- | --- |
|  | Value | df | Asymptotic Significance (2-sided) | Exact Sig. (2-sided) | Exact Sig. (1-sided) |
| Pearson Chi-Square | ,157^a^ | 1 | ,692 |  |  |
| Continuity Correction^b^ | ,000 | 1 | 1,000 |  |  |
| Likelihood Ratio | ,165 | 1 | ,684 |  |  |
| Fisher's Exact Test |  |  |  | 1,000 | ,575 |
| Linear-by-Linear Association | ,157 | 1 | ,692 |  |  |
| N of Valid Cases | 265 |  |  |  |  |

| a. 2 cells (50,0%) have expected count less than 5. The minimum expected count is 1,37. |
| --- |
| b. Computed only for a 2x2 table |

**SS(1), normal (0) * mutace SHOX(X)**

| **Crosstab** | | | | | |
| --- | --- | --- | --- | --- | --- |
|  | | | mutace SHOX(X) | | Total |
|  |  |  | 0 | 1 |  |
| Count | SS(1), normal (0) | 0 | 90 | 1 | 91 |
|  |  | 1 | 171 | 3 | 174 |
|  | Total | | 261 | 4 | 265 |
| % within SS(1), normal (0) | SS(1), normal(0) | 0 | 98,9% | 1,1% | 100,0% |
|  |  | 1 | 98,3% | 1,7% | 100,0% |
|  | Total | | 98,5% | 1,5% | 100,0% |

| **Chi-Square Tests** | | | | | |
| --- | --- | --- | --- | --- | --- |
|  | Value | df | Asymptotic Significance (2-sided) | Exact Sig. (2-sided) | Exact Sig. (1-sided) |
| Pearson Chi-Square | ,157^a^ | 1 | ,692 |  |  |
| Continuity Correction^b^ | ,000 | 1 | 1,000 |  |  |
| Likelihood Ratio | ,165 | 1 | ,684 |  |  |
| Fisher's Exact Test |  |  |  | 1,000 | ,575 |
| Linear-by-Linear Association | ,157 | 1 | ,692 |  |  |
| N of Valid Cases | 265 |  |  |  |  |

| a. 2 cells (50,0%) have expected count less than 5. The minimum expected count is 1,37. |
| --- |
| b. Computed only for a 2x2 table |

**RR(1), normal (0) * dup SHOX(X)**

| **Crosstab** | | | | | |
| --- | --- | --- | --- | --- | --- |
|  | | | dup SHOX(X) | | Total |
|  |  |  | 0 | 1 |  |
| Count | RR(1), normal (0) | 0 | 88 | 3 | 91 |
|  |  | 1 | 173 | 1 | 174 |
|  | Total | | 261 | 4 | 265 |
| % within SS(1), normal(0) | SS(1), normal(0) | 0 | 96,7% | 3,3% | 100,0% |
|  |  | 1 | 99,4% | 0,6% | 100,0% |
|  | Total | | 98,5% | 1,5% | 100,0% |

| **Chi-Square Tests** | | | | | |
| --- | --- | --- | --- | --- | --- |
|  | Value | df | Asymptotic Significance (2-sided) | Exact Sig. (2-sided) | Exact Sig. (1-sided) |
| Pearson Chi-Square | 2,978^a^ | 1 | ,084 |  |  |
| Continuity Correction^b^ | 1,428 | 1 | ,232 |  |  |
| Likelihood Ratio | 2,801 | 1 | ,094 |  |  |
| Fisher's Exact Test |  |  |  | ,119 | ,119 |
| Linear-by-Linear Association | 2,967 | 1 | ,085 |  |  |
| N of Valid Cases | 265 |  |  |  |  |

| a. 2 cells (50,0%) have expected count less than 5. The minimum expected count is 1,37. |
| --- |
| b. Computed only for a 2x2 table |

**SS(1), normal (0) * del regul SHOX (X)**

| **Crosstab** | | | | | |
| --- | --- | --- | --- | --- | --- |
|  | | | del regul SHOX (X) | | Total |
|  |  |  | 0 | 1 |  |
| Count | SS(1), normal(0) | 0 | 90 | 1 | 91 |
|  |  | 1 | 172 | 2 | 174 |
|  | Total | | 262 | 3 | 265 |
| % within SS(1), normal(0) | SS(1), normal (0) | 0 | 98,9% | 1,1% | 100,0% |
|  |  | 1 | 98,9% | 1,1% | 100,0% |
|  | Total | | 98,9% | 1,1% | 100,0% |

| **Chi-Square Tests** | | | | | |
| --- | --- | --- | --- | --- | --- |
|  | Value | df | Asymptotic Significance (2-sided) | Exact Sig. (2-sided) | Exact Sig. (1-sided) |
| Pearson Chi-Square | ,001^a^ | 1 | ,971 |  |  |
| Continuity Correction^b^ | ,000 | 1 | 1,000 |  |  |
| Likelihood Ratio | ,001 | 1 | ,970 |  |  |
| Fisher's Exact Test |  |  |  | 1,000 | ,728 |
| Linear-by-Linear Association | ,001 | 1 | ,971 |  |  |
| N of Valid Cases | 265 |  |  |  |  |

| a. 2 cells (50,0%) have expected count less than 5. The minimum expected count is 1,03. |
| --- |
| b. Computed only for a 2x2 table |

**SS(1), normal (0) * del regul SHOX (Y)**

| **Crosstab** | | | | | |
| --- | --- | --- | --- | --- | --- |
|  | | | del regul SHOX (Y) | | Total |
|  |  |  | 0 | 1 |  |
| Count | SS(1), normal (0) | 0 | 88 | 3 | 91 |
|  |  | 1 | 174 | 0 | 174 |
|  | Total | | 262 | 3 | 265 |
| % within SS(1), normal(0) | SS(1), normal (0) | 0 | 96,7% | 3,3% | 100,0% |
|  |  | 1 | 100,0% | 0,0% | 100,0% |
|  | Total | | 98,9% | 1,1% | 100,0% |

| **Chi-Square Tests** | | | | | |
| --- | --- | --- | --- | --- | --- |
|  | Value | df | Asymptotic Significance (2-sided) | Exact Sig. (2-sided) | Exact Sig. (1-sided) |
| Pearson Chi-Square | 5,802^a^ | 1 | ,016 |  |  |
| Continuity Correction^b^ | 3,230 | 1 | ,072 |  |  |
| Likelihood Ratio | 6,479 | 1 | ,011 |  |  |
| Fisher's Exact Test |  |  |  | ,040 | ,040 |
| Linear-by-Linear Association | 5,780 | 1 | ,016 |  |  |
| N of Valid Cases | 265 |  |  |  |  |

| a. 2 cells (50,0%) have expected count less than 5. The minimum expected count is 1,03. |
| --- |
| b. Computed only for a 2x2 table |

**SS(1), normal (0) * dup regul SHOX**

| **Crosstab** | | | | | |
| --- | --- | --- | --- | --- | --- |
|  | | | dup regul SHOX | | Total |
|  |  |  | 0 | 1 |  |
| Count | SS(1), normal(0) | 0 | 87 | 4 | 91 |
|  |  | 1 | 164 | 10 | 174 |
|  | Total | | 251 | 14 | 265 |
| % within SS(1), normal(0) | SS(1), normal(0) | 0 | 95,6% | 4,4% | 100,0% |
|  |  | 1 | 94,3% | 5,7% | 100,0% |
|  | Total | | 94,7% | 5,3% | 100,0% |

| **Chi-Square Tests** | | | | | |
| --- | --- | --- | --- | --- | --- |
|  | Value | df | Asymptotic Significance (2-sided) | Exact Sig. (2-sided) | Exact Sig. (1-sided) |
| Pearson Chi-Square | ,218^a^ | 1 | ,640 |  |  |
| Continuity Correction^b^ | ,032 | 1 | ,859 |  |  |
| Likelihood Ratio | ,224 | 1 | ,636 |  |  |
| Fisher's Exact Test |  |  |  | ,777 | ,441 |
| Linear-by-Linear Association | ,217 | 1 | ,641 |  |  |
| N of Valid Cases | 265 |  |  |  |  |

| a. 1 cells (25,0%) have expected count less than 5. The minimum expected count is 4,81. |
| --- |
| b. Computed only for a 2x2 table |
